# Thinking about Believing: Can Metacognitive Reflection Encourage Belief Updating?

**DOI:** 10.3390/jintelligence12050047

**Published:** 2024-04-28

**Authors:** Allison P. O’Leary, Wesley Fletcher

**Affiliations:** Department of Psychology, Brevard College, Brevard, NC 28712, USA; wesflet@gmail.com

**Keywords:** metacognition, metacognitive reflection, belief updating, belief change

## Abstract

People often cling to their beliefs even in the face of counterevidence. The current study explored metacognitive reflection as a potential driver for belief updating. In a randomized controlled experiment (*n* = 155), participants rated their degree of agreement with a statement regarding genetic modification in humans. Following this, participants were presented with a passage containing an argument counter to their indicated belief. Participants in the metacognition condition were asked to deeply reflect on the ways in which the passage was similar to or different from their current beliefs. Participants in the control condition were asked to engage in more shallow reflection on the composition of the passage. After reflecting on the counterevidence, participants were asked to again rate their agreement with the statement regarding human gene modification. Both groups updated their initial beliefs to be more consistent with the presented counterevidence. Although greater belief updating was observed in those who metacognitively reflected on the passage, this effect did not reach significance (*p* = .055). These findings suggest that reflecting on counterevidence has the potential to encourage belief updating, regardless of whether that reflection is metacognitive in nature, and provide promise for future work investigating the role of metacognition in belief updating.

## 1. Introduction

We often encounter information that runs counter to our existing beliefs. For example, during the COVID-19 pandemic, someone who believed the new COVID-19 vaccines were ineffective and unsafe may have encountered statistics speaking to the vaccine’s extensive testing, low risk of serious side effects, and high effectiveness at preventing hospitalization (Centers for Disease Control and Prevention 2023). When people encounter information inconsistent with their beliefs, how likely are they to update those beliefs? Previous findings are mixed, with some showing that people sometimes cling to their beliefs (Mandelbaum 2014; Nickerson 1998; Kaplan et al. 2016), or even intensify their beliefs (Nyhan and Reifler 2010; Nyhan et al. 2014), despite counterevidence. Other studies show that people will revise their beliefs to take the counterevidence into account (Kardash and Scholes 1995; Slusher and Anderson 1996; Anglin 2019).

The questions of whether and how people update their beliefs in the face of counterevidence have significant implications for human intelligence, scientific progress, public policy, and personal behaviors. To build a growing collection of scientific knowledge, we must revise theories and predictions in light of new information. As a society, it is crucial that we update policies in line with scientific advancement. As individuals, it is important that we update our beliefs and our behaviors based on new information. Although most of the research on belief change has focused on the role of persuasive messaging (Jaccard 1981), the extent to which we update beliefs may depend on how thoroughly we *engage* with the presented evidence (Petty and Briñol 2008). The primary goal of this research was to investigate whether reflecting on information counter to one’s beliefs would encourage belief updating. In addition, would engaging more deeply with the counterevidence by comparing it with existing beliefs (what we refer to as *metacognitive reflection*) encourage belief change?

### 1.1. Counterevidence and Belief Change

As stated above, previous work has yielded mixed findings regarding the role of counterevidence in belief updating. Some work has shown that people cling to or even intensify beliefs in the face of counterevidence—a phenomenon referred to as belief perseverance (Walster et al. 1967; Ross et al. 1975). There are many purported mechanisms for this effect. People may minimize or discount conflicting evidence (Kaplan et al. 2016), critically evaluate the evidence opposing their beliefs (Lord et al. 1979), or generate explanations that support their original belief (Chan et al. 2017).

Other work has shown that people successfully update their beliefs when presented with counterevidence (Kardash and Scholes 1995; Slusher and Anderson 1996; Anglin 2019). In one study, researchers measured participants’ political and non-political beliefs before and after encountering evidence counter to these beliefs (Kaplan et al. 2016). Although the strength of participants’ beliefs decreased across both belief types, participants were more likely to update their non-political beliefs. Others have similarly theorized that beliefs with which people self-identify, like political or religious beliefs, are more resistant to change (Porot and Mandelbaum 2020).

Explanations regarding these mixed findings often describe differences in the ways participants interacted with the presented counterevidence. For example, Kardash and Scholes (1995) suggested that persuasive messages were more likely to lead to belief updating if those messages encouraged people to (1) consider explanations in conflict with their existing beliefs or (2) think deeply about an issue. In addition, Slusher and Anderson (1996) emphasized the role of elaborative interrogation, or the ability for people to generate their own explanations for a fact or finding, arguing that this process accounted for belief updating in their study. Based on these findings, deeply reflecting on the counterevidence, including how it relates to one’s existing beliefs, may encourage belief updating.

### 1.2. Metacognition and Belief Change

Metacognition is often defined as thinking about thinking or knowing about knowing, and is typically described as having two subcomponents: metacognitive monitoring (or metacognitive knowledge) and metacognitive control (or metacognitive regulation; Flavell 1979). Whereas metacognitive monitoring describes the ability to assess and evaluate one’s knowledge or performance, metacognitive control describes the ability to update one’s behavior in line with one’s goals. These processes intimately interact, yet appear to develop independently of one another (O’Leary and Sloutsky 2017; O’Leary and Sloutsky 2019). Engaging in metacognition can encourage individuals to reflect on what they know, what they are still questioning, and how they can improve. Additionally, it allows individuals to develop strategies for how to *apply* the information they are learning (Wood 2009; Tanner 2012). As such, many studies have demonstrated a positive relation between metacognitive ability and academic achievement (e.g., Vrugt and Oort 2008; Young and Fry 2008).

One way to encourage metacognition is through promoting *metacognitive reflection*. John Dewey famously suggested that we learn more from reflecting on experience than we do from the experience itself (Dewey 1933). Empirical work has shown that encouraging students to metacognitively reflect on what they are learning can improve their understanding of that material (Ellis 2001; Ellis and Denton 2010; Baliram and Ellis 2019). For example, Mevarech and Kramarski (1997) found that training that provided students with metacognitive questioning during learning (i.e., asking students questions like “What are the differences between the problem at hand and the previous problem?”) improved students’ mathematics performance compared to a control group that received no training.

Although the impact of metacognitive reflection on learning is clear, there is little research investigating the role of metacognitive reflection in belief updating. Some research has assessed metacognition *following* belief change (i.e., how aware individuals are that their beliefs have changed). For example, Wolfe and Williams (2018) found that participants who updated their beliefs after encountering counterevidence were unaware that their beliefs had changed. In fact, their recollections of their initial beliefs were biased toward their current beliefs, reflecting counterevidence-consistent memory updating in the face of new evidence despite poor metacognitive awareness of this change. Although this study reflects poor metacognition following belief change, the role of metacognitive reflection as a *driver* of belief change remains largely uninvestigated. We predict that individuals prompted to deeply reflect on presented counterevidence and how it compares to existing beliefs would be more likely to update their beliefs than those who did not engage in this type of reflection.

### 1.3. Individual Differences in Metacognition and Belief Change

Metacognition is thought to be an ability that varies across individuals. For example, previous work has shown that individuals’ scores on the Metacognitive Awareness Inventory (MAI) varied substantially and were associated with individual differences in end of course grades and grade point averages (Young and Fry 2008). The MAI is a 52-item self-report survey that measures individuals’ metacognitive knowledge (monitoring) and metacognitive regulation (control) as they pertain to learning (Schraw and Dennison 1994; Harrison and Vallin 2017). The *metacognitive knowledge* portion of the inventory is further divided into declarative, procedural, and conditional knowledge subcategories, which largely assess an individual’s knowledge of how, when, and why to use learning strategies. The *regulation of cognition* portion is subdivided into planning, comprehension monitoring, information management strategies, debugging, and evaluation skills subcategories, which measure an individual’s control over their learning. In the current study, we aimed to evaluate whether metacognitively reflecting on counterevidence would increase individuals’ likelihood of updating their beliefs. As such, it was important to account for individual differences in metacognitive ability. Perhaps those high in metacognitive ability would be more likely to update their beliefs regardless of whether they were asked to metacognitively reflect (i.e., they may do so spontaneously). Previous work suggests that individuals’ metacognitive sensitivity was linked to their belief updating. For example, the beliefs of individuals with greater metacognitive sensitivity were less likely to be influenced by climate change misinformation (Fischer et al. 2022). In the current study, we measured individual differences in metacognitive ability using the MAI to account for this possibility.

It is also possible that other individual differences could impact individuals’ metacognition and/or their tendency to update their beliefs. For example, people with polarized political beliefs (regardless of political orientation) show lower levels of cognitive flexibility, reflecting more difficulty shifting perspective (Zmigrod et al. 2020). In addition, those who score higher on measures of right-wing authoritarianism are less likely to update their beliefs when presented with challenges to those beliefs (Sinclair et al. 2020). Rollwage et al. (2018) found that participants with more radical political beliefs showed less metacognitive sensitivity in a perceptual discrimination task. In addition, they were less likely to update their confidence estimates when provided with feedback on their task performance, indicating difficulty updating beliefs in the face of counterevidence. These findings point to an additional link between metacognition and belief change, in that individuals who hold strong political beliefs may be less likely to use metacognitive processes to update their beliefs. Additionally, Said et al. (2021) found that greater metacognitive sensitivity was linked to a lower likelihood of polarized climate change beliefs, but this link was not found for beliefs about nanotechnology (a less-politicized topic). This suggests that metacognition may play a unique role in driving belief updating for political topics. In the current study, we measured participants’ political beliefs to assess their relation to metacognition and belief change.

### 1.4. The Current Study

The current study had two primary aims. The first was to examine whether individuals would update their beliefs in the face of counterevidence. The second aim was to determine whether individuals would be more likely to update their beliefs when asked to *metacognitively reflect* on the counterevidence, as opposed to engaging in more shallow reflection.

In the current study, we took an experimental approach to test whether individuals updated their beliefs after encountering counterevidence. We measured participants’ beliefs about human gene editing (a topic on which people are generally divided; Riggan et al. 2019) before and after presenting information counter to their initial beliefs. Once presented with this counterevidence, we manipulated how participants were prompted to engage with this new information. Participants in the *metacognition* condition were asked to deeply reflect on the ways in which the counterevidence was consistent or inconsistent with their initial beliefs. Participants in the *control* condition were asked to reflect on surface-level qualities of the counterevidence, like the grammar and composition of the counterevidence passage (see Table 1). Following the counterevidence and reflection, participants were again asked to indicate their beliefs on the topic of human gene editing.

Because of the body of work highlighting resistance to belief updating, we predicted that participants in the metacognition condition would update their beliefs in the face of counterevidence and that participants in the control condition would not. Previous work suggested that individuals are more likely to update their beliefs if they deeply consider and generate ideas about conflicting information (Kardash and Scholes 1995; Slusher and Anderson 1996). Engaging in this kind of metacognitive reflection should encourage individuals to consider ways their current beliefs are compatible and incompatible with new, conflicting information, which may lead them to update their position. We also measured individual differences in metacognitive ability (using the MAI) and political orientation to examine whether individual differences in belief updating could be attributed to these factors. We expected to see a positive relation between belief change and scores on the MAI and a positive relation between belief change and political polarization.

## 2. Materials and Methods

### 2.1. Participants

This study included a total of 155 participants (107 male, 43 female, 4 non-binary, and 1 other) recruited from an introductory psychology course at a small liberal arts college. Participants were undergraduate students aged between 19 and 31 (*M* = 20, *SD* = 1.97) enrolled in an introductory psychology class, who received course credit for their participation. This study was approved by the Institutional Review Board at Brevard College (protocol number: 21-22.04). Prior to the study, all participants were informed about the study’s procedure and provided informed consent. The experiment was administered by the researchers, who visited each introductory psychology classroom. Participants were randomly assigned to the control (*n* = 78) or metacognition (*n* = 77) conditions.

### 2.2. Materials

#### 2.2.1. Metacognitive Awareness Inventory (MAI)

The metacognitive awareness inventory (MAI) is a 52-item survey used to measure individual differences in the following two categories of metacognition: knowledge of cognition and regulation of cognition (Schraw and Dennison 1994). In the MAI, each of these two categories are further broken down into subcomponents. The items used to measure knowledge of cognition included declarative knowledge, procedural knowledge, and conditional knowledge subcomponents. The items used to measure the regulation of cognition included the following subcomponents: planning, information management strategies, comprehension monitoring, debugging strategies, and evaluation. Each item included a statement and participants were asked to indicate whether each statement was true or false. Some items used to measure knowledge of cognition included “I am a good judge of how well I understand something” (declarative knowledge) and “I try to use strategies that have worked in the past” (procedural knowledge). Example items used to measure the regulation of cognition included “I focus on the meaning and significance of new information” (information management strategies) and “I re-evaluate my assumptions when I get confused” (debugging strategies; Schraw and Dennison 1994).

#### 2.2.2. Belief Statement

We conducted a pilot study to identify a topic for which participants would hold diverse views and to ensure that the supportive and critiquing counterevidence passages were similarly persuasive. In the pilot, we measured beliefs regarding the following 3 topics: human gene editing, the utility of space exploration, and physician-assisted suicide. We also assessed individuals’ sensitivity to both the supportive and critiquing counterevidence passages for each topic. For human gene editing, we found an even split between those who initially agreed or disagreed with the topic idea, and we found that participants were similarly persuaded by both the supportive and critiquing counterevidence passages we developed; thus, we selected this topic for use in the study proper.

In the study proper, participants were shown the belief statement “Scientists should be able to modify human DNA to control the expression of physical traits and risk of disease” during both pre- and post-test and were asked to rate their level of agreement on a 1 to 6 Likert scale.

#### 2.2.3. Counterevidence Passages

Counterevidence passages were developed to persuade participants to challenge their currently held beliefs. If participants disagreed with the belief statement (selected strongly disagree, somewhat disagree, or slightly disagree), they were presented with a passage in support of human gene editing. If participants agreed with the belief statement (selected slightly agree, somewhat agree, or strongly agree), they were presented with a passage critiquing the idea of human gene editing. Participants read only one of these passages, and the two passages were designed to be similar in length and complexity. Both passages began with an attention-grabbing opening sentence and some background information on human genetic editing: “As technology advances, what may have been called science fiction a few decades ago may have moved into the realm of possibility. CRISPR is a genetic editing tool that gives humans the ability to splice out or even replace specific genes”.

Following this introduction, the **supportive** passage continued, “Genes that contribute to cancer or other diseases could potentially be spliced out of the genome of human embryos and eliminate serious genetic disease in our society. This technology shows great promise, as it has already been used to address diseases like HIV and sickle-cell anemia. Imagine all the good that this new technology can do! This technology could increase the average human lifespan and help create a healthier society. This is a tool that has the potential to change the world for the better”. Following the introduction, the **critiquing** passage read, “At first glance this seems like a really cool piece of technology, but is it ethical? With this technology, genes associated with certain traits such as hair and skin color could potentially be targeted and altered. In theory, people could create “designer babies” with all their traits selected by the parents. The risk of this technology to create “superior” human beings must not be taken lightly. The Nazi regime during WWII used physical traits to determine the value of human life. Imagine if they had access to gene editing technology. This technology needs to be regulated and the implications of research involving CRISPR should be heavily considered”.

#### 2.2.4. Reflective Prompts

Participants were randomly assigned to the control or metacognition condition, which determined the reflective prompts they would receive following the presentation of the counterevidence passages. Participants in both conditions were given an equal number of reflective questions, including 2 Likert scale and 4 open-ended questions in each condition. Participants in the **metacognition condition** were asked to metacognitively reflect on how the new information in the counterevidence passage interacted with their existing beliefs. Each of these prompts recruited metacognitive processes because participants were asked to not only consider the beliefs themselves but also their thoughts about their beliefs (at a meta-level). First, participants were asked to consider how their beliefs compared and contrasted with the new information just presented, reflecting on the similarity and novelty of this new information relative to their existing beliefs. In addition, the metacognitive prompts encouraged participants to reflect not only on the content of the argument but also on its quality and strength. Finally, they were asked to consider how convincing this new information was and why. Participants in the **control condition** were asked to reflect on surface features of the counterevidence passage like the grammar and composition of the passage. The control condition was designed to control for time spent reflecting on and interacting with the passage, providing exposure to the counterevidence in a way that does not engage deeper metacognitive processing. All reflective prompts can be found in Table 1.

### 2.3. Design

This study employed a mixed design, with one within-subject factor (time point: pre-test belief rating and post-test belief rating) and two between-subject factors (counterevidence type: supportive vs. critiquing; condition: control vs. metacognition).

### 2.4. Procedure

#### 2.4.1. Demographics and MAI

Participants completed the experiment during their introductory psychology class, using Qualtrics on their personal devices. Participants were first asked to respond to several demographic questions, including year of birth, year in college (freshman, sophomore, junior, or senior), gender, whether they speak more than one language, political identity (from very liberal to very conservative), and racial identity. Following this, participants completed the 52-item MAI to measure individual differences in knowledge and regulation of cognition.

#### 2.4.2. Pre-Test

In the pre-test phase, participants were asked to indicate their level of agreement with the following belief statement: “Scientists should be able to modify human DNA to control the expression of physical traits and risk of disease”. Participants were asked to indicate their level of agreement on a 6-point Likert scale. As some level of agreement or disagreement was critical in determining the type of counterevidence to present, we included an even number of options to prevent fence-sitting.

#### 2.4.3. Counterevidence and Reflection

Following the pre-test, participants were presented with a counterevidence passage containing an argument counter to their just-indicated belief. As such, the counterevidence passage presented depended on each participant’s level of agreement with the belief statement in the pre-test phase. Participants who initially disagreed with the belief statement (i.e., selected an agreement rating of strongly disagree, somewhat disagree, or slightly disagree) were presented with a passage **supportive** of human gene editing. Participants who agreed with the belief statement on the pre-test (selected an agreement rating of slightly agree, somewhat agree, or strongly agree) were presented with a passage **critiquing** the idea of human gene editing.

After reading the counterevidence passage, participants were presented with reflective prompts based on their assigned condition (control or metacognition condition). Participants in the **metacognition condition** responded to questions that encouraged them to reflect on the ways the passage was consistent and inconsistent with their existing beliefs, the quality of the author’s argument, and the parts of the passage that were compelling or convincing. Participants in the **control condition** responded to questions that encouraged reflection on the passage’s grammar and composition (see Table 1). Participants in both conditions were required to respond to each of the reflection prompts before being allowed to proceed to the post-test phase. This was done to ensure that participants in both conditions spent a similar amount of time interacting with the counterevidence passage.

#### 2.4.4. Post-Test

In the post-test phase, participants were asked to rate their level of agreement with the same belief statement presented at pre-test: “Scientists should be able to modify human DNA to control the expression of physical traits and risk of disease”. Again, participants were asked to indicate their level of agreement on a 6-point Likert scale. Belief updating was measured by assessing the difference between participants’ belief ratings from pre-test to post-test.

## 3. Results

The aim of the current study was to investigate whether providing participants with counterevidence would encourage them to update their beliefs. We expected participants in the metacognition condition (who reflected on how the counterevidence related to their existing beliefs) to update their beliefs and those in the control condition to show no change in belief. Because the presented counterevidence depended on participant beliefs assessed at pre-test, participants who received the supportive counterevidence may have updated their beliefs to agree with the pre-test belief statement *more*, whereas those who received the critiquing counterevidence may have updated their beliefs to agree *less*. If this were the case, averaging raw scores across individuals would have canceled out any changes from pre- to post-test. To prevent this, we transformed participants’ pre- and post-test belief scores. Responses of slightly agree, somewhat agree, and strongly agree were re-coded to be the same as responses of slightly disagree, somewhat disagree, and strongly disagree, respectively. If a participant’s pre-test scores were re-coded, their post-test scores were re-coded in the same fashion. This transformation ensured that changes from pre-test to post-test would demonstrate counterevidence-consistent changes in belief, regardless of participants’ initial level of agreement with the belief statement. We also computed a belief change variable that measured the counterevidence-consistent change in belief from pre- to post-test.

At pre-test, 51% of participants indicated agreement with the belief statement (chose slightly agree, somewhat agree, or strongly agree), and 49% of participants indicated disagreement (chose slightly disagree, somewhat disagree, or strongly disagree). We examined the percentage of individuals who showed no change in belief, those who updated their belief to align with counterevidence, and those who strengthened their initial beliefs. In the control condition, 62% of participants showed no change in belief, 35% of participants had a counterevidence-consistent change in belief, and 4% strengthened their initial beliefs. In the metacognition condition, 52% showed no change in belief, 43% had a counterevidence-consistent change in belief, and 5% strengthened their initial beliefs.

**Figure 1:**
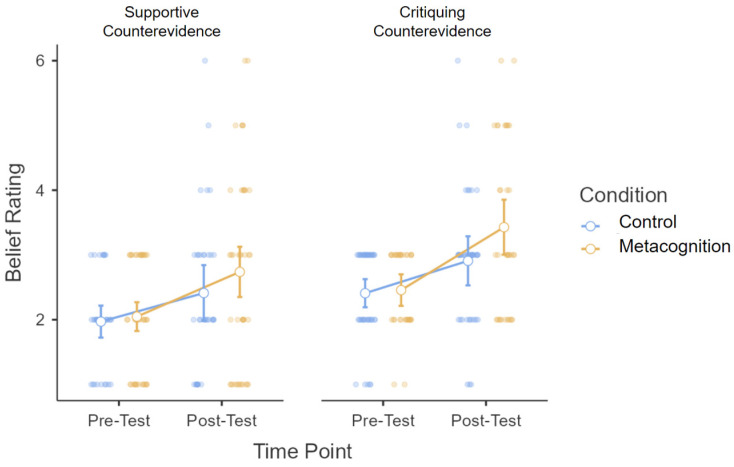
Belief updating as a function of time point, counterevidence type, and condition. Note: Increases from pre- to post-test reflect an increase in counterevidence-consistent belief. Dots represent individual data points, and error bars represent standard error.

For our main analysis, we conducted a mixed model ANOVA on participants’ belief ratings with time point (pre-test belief vs. post-test belief) as a within-subjects factor and counterevidence type (supportive vs. critiquing) and condition (control vs. metacognition) as between-subjects factors. Our main hypothesis predicted a significant Time X Condition interaction, in that participants in the metacognition condition would demonstrate greater belief change from pre-test to post-test than participants in the control condition. The mean difference between pre-test and post-test was 0.82 (*SD* = 1.40) in the metacognition condition and 0.47 (*SD* = 0.85) in the control condition. However, the Time X Condition interaction did not reach significance, *F*(1, 151) = 3.73, *p* = .055, η_p_^2^ = 0.024 (see Figure 1). No other interactions were significant (all *p*s > 0.3). Across timepoints, the mean belief rating was 2.67 (*SD* = 0.87) in the metacognition condition and 2.43 (*SD* = 0.87) in the control condition. However, the main effect of condition was not significant, (*F*(1, 151) = 3.04, *p* = .08, η_p_^2^ = 0.02).

There was a main effect of time point (*F*(1, 151) = 48.62, *p* < .001, η_p_^2^ = 0.24), reflecting a significant increase in the counterevidence-consistent belief from pre-test (*M* = 2.23, *SD* = 0.75) to post-test (*M* = 2.87, *SD* = 1.31). Surprisingly, post-hoc paired-sample *t*-tests showed that participants in both the metacognition condition (*t*(76)= −5.15, *p* < .001, *d* = 0.59) and the control condition (*t*(77)= −4.94, *p* < .001, *d* = 0.56) significantly updated their beliefs from pre-test to post-test.

Unexpectedly, there was also a significant main effect of counterevidence type, *F*(1, 151) = 13.36, *p* < .001, η_p_^2^ = 0.08. Those who received critiquing counterevidence reported a greater counterevidence-consistent belief. We believe that this result is due to the fact that, even at pre-test (before any counterevidence was presented), those who received critiquing counterevidence (and who indicated initial agreement with the belief statement; *M* = 2.43, *SD* = 0.65) demonstrated a greater counterevidence-consistent belief than those who received supportive counterevidence (and who indicated initial disagreement; *M* = 2.01, *SD* = 0.79; *t*(153) = −3.58, *p* < .001, *d* = 0.58). In other words, the level of agreement reported at pre-test (in those participants who would later receive critiquing counterevidence) was stronger than the level of disagreement reported at pre-test (in those participants who would receive supportive counterevidence). Thus, this put the two groups at a different starting point. Although this finding was unexpected, we were not particularly concerned about this difference because our main analysis investigated *changes* in belief following exposure to counterevidence. Although a higher pre-test score may have restricted the amount of change possible at post-test, we did not see this play out in our data. Post-test scores in those who received critiquing counterevidence were far from ceiling.

We also investigated whether the strength of participants’ beliefs at pre-test was related to the extent to which they updated their belief. There was no correlation between pre-test scores and belief change (counterevidence-consistent belief updating from pre- to post-test; *r* = −0.11, *p* = .15), indicating that those with beliefs on either end of the Likert scale were no more or less likely to update their beliefs. This was true for participants in both the metacognition and control conditions (both *p*s > 0.09).

To determine whether individuals’ belief change was related to individual differences in metacognitive ability, we assessed the relation between belief change and the components of metacognition measured by the MAI. The mean amount of belief change across participants was 0.65 (*SD* = 1.16). There were no significant correlations between belief change and knowledge of cognition (*r* = 0.002, *p* = .98) or regulation of cognition (*r* = 0.09, *p* = .25), nor any of the subcomponents within each of those categories (all *p*s > 0.11). The lack of relation between belief change, knowledge of cognition, and regulation of cognition held true for participants in both the metacognition condition and the control condition (all *p*s > 0.08).

We also assessed the relation between belief change and political affiliation. Participants rated their political affiliation on a 1–5 Likert scale, with 1 being very liberal and 5 being very conservative (*M* = 3.24, *SD* = 1.06). We also evaluated the strength of participants’ political affiliation, with 1 being moderate, 2 being somewhat liberal or conservative, and 3 being very liberal or conservative (*M* = 1.75, *SD* = 0.78). The extent of participants’ belief change was not associated with participants’ political affiliation (*r* = −0.05, *p* = .52), nor was it associated with the *strength* of their political affiliation (*r* = −0.11, *p* = .17).

We also investigated whether participants’ *engagement* with the counterevidence reflection prompt differed by condition or was related to belief updating. Participants in each condition received four open-ended questions (see Table 1). We totaled the number of characters written in each participants’ responses to these four questions and used this as a measure of engagement with the reflection prompts. We excluded two participants from this analysis who only entered one-character responses for each question (either “.” or “k”) in an attempt to bypass these required questions. Participants in the metacognition condition (*M* = 345, *SD* = 281) wrote significantly more characters than those in the control condition (*M* = 275, *SD* = 232; *t*(151) = −2.02, *p* < .05, *d* = 0.33), indicating that they engaged more deeply with the reflective prompts. However, the length of participants’ responses was not significantly correlated with the extent to which they updated their beliefs (*r* = −0.02, *p* = .83).

## 4. Discussion

The current study investigated the process of belief updating by measuring participants’ beliefs about human gene editing before and after exposure to an argument counter to their initial beliefs. To our knowledge, this is the first study to present targeted counterevidence based on participants’ beliefs. Our primary interest was whether deep, metacognitive reflection on that counterevidence would encourage belief updating more than shallow reflection. Though we expected to see belief updating in the metacognition condition only, we found that participants updated their beliefs both when shallowly reflecting on the counterevidence passage (i.e., reflecting on its composition and grammar) and when more deeply reflecting on the counterevidence (i.e., reflecting on its similarities and differences with existing beliefs). The amount of belief updating in the metacognition condition was numerically larger than that in the control condition, reflecting a greater impact of metacognitive reflection. However, this difference did not reach significance (*p* = .055; see Figure 1).

These data provide evidence that exposure to and reflection on a single piece of counterevidence can induce belief updating. This is in contrast with work showing that beliefs are often resistant to change, even in the face of counterevidence (Scheffer et al. 2022). Our findings are consistent with work showing that individuals can successfully update their beliefs after encountering evidence inconsistent with their initial position. For example, Anglin (2019) found that participants updated their beliefs on religion, politics, gun control, and capital punishment following exposure to research summaries on those topics. In this study, the authors attributed participants’ belief updating to the quality of evidence presented. Indeed, much of the work on belief updating has focused on the role of persuasive arguments (Petty and Cacioppo 1986). In the current study, however, we aimed to manipulate the level of *engagement* with persuasive counterevidence to measure its effects on belief updating.

Though the interaction between timepoint and condition did not reach significance (*p* = .055; see Figure 1), our data highlight an important trend suggesting that deeper metacognitive reflection may have a greater impact on belief updating than more shallow reflection. In addition to this finding, we provided some evidence that participants did more deeply process the counterevidence in the metacognition condition. For example, participants’ responses to the open-ended reflection prompts were longer than those in the control condition. Together, these findings leave open the possibility that metacognitive reflection can encourage belief updating. There are a few possible mechanisms through which metacognitive reflection may influence belief updating. First, metacognitive reflection may minimize confirmation bias by encouraging deeper processing of information inconsistent with one’s beliefs. For example, reflecting on how one’s current beliefs compare and contrast with a new and counter viewpoint make it more difficult to downweigh the evidence against one’s position (Rollwage and Fleming 2021). In addition to and compatible with the above explanation, metacognitive reflection may increase one’s metacognitive sensitivity (the ability to judge the correctness of one’s belief). Metacognitive reflection may lead one to more precisely identify uncertainty in their own beliefs, prompting one to evaluate new information more carefully and to process the counterevidence more deeply (Rollwage et al. 2018).

Although we originally expected participants to update their beliefs only following metacognitive reflection, even shallow engagement with the counterevidence was enough to prompt belief updating (though to a seemingly lesser extent). Why did participants update their beliefs even when prompted to shallowly reflect on the passage’s grammar and composition? One explanation is that reflecting on the surface-level features of a passage provided sufficient opportunity to increase participants’ familiarity fluency with the passage. For example, participants in the control condition answered questions like “Please briefly describe what element(s) of this passage would be hardest for English learning individuals to comprehend” and “How could the grammar in this passage be modified for English learners to make it more understandable?”. Answering these questions may have required multiple close readings of the passage. It is well known that multiple exposures to information increase processing fluency, which can be interpreted as a signal of truth (Hassan and Barber 2021). This process, rather than metacognitive reflection, may be responsible for participants’ belief updating in the control condition.

Another explanation is that participants engaged in deep, metacognitive reflection in both conditions. Although the reflective prompts in the control condition were meant to encourage shallow reflection, some of the questions may have encouraged participants to consider the counterevidence more deeply. For example, participants were asked, “How well written is this passage?”. Though participants were presented this question in the context of other questions about the passage’s grammatical structure and understandability, it may have been interpreted as referring to the quality of the passage’s argument. This may have encouraged deeper reflection about the content of the passage. The current dataset cannot determine the mechanism through which participants updated their beliefs in the control condition, but these findings raise new and interesting questions about the role of depth of reflection in belief change. Future work should include a pure control condition, with no reflective prompts, to determine the extent to which reflection per se plays a role in belief change.

Yet another less interesting explanation for belief updating in the control condition is that participants in both conditions engaged in hypothesis guessing. Perhaps exposure to counterevidence prompted participants to speculate that the purpose of the study was to update their beliefs. This may have led participants to reactively modify their responses to be in line with the researchers’ expectations. Despite this possibility, the majority of participants showed no change in belief (62% in the control condition and 52% in the metacognition condition). This possibility could be directly addressed in future work by asking participants to indicate their beliefs about the study’s purpose following the experiment.

It is worth noting that we chose the topic of human gene editing because it was not a strongly partisan, politicized topic. This was because we wanted to test the effects of metacognitive reflection per se and wanted to minimize participants’ self-identification with the topic. However, recent work by Said et al. (2021) raised the possibility that there may be a stronger link between metacognition and belief updating for more politically polarizing topics, which may explain why we did not observe a larger difference in belief updating between the metacognition and control conditions. These researchers found that those with greater metacognitive sensitivity were less likely to develop polarized beliefs about climate change but not nanotechnology, reasoning that there may be a stronger link between metacognition and belief updating for topics where individuals initially held strong beliefs. Perhaps for entrenched beliefs, there is a greater need for metacognitive reflection in the belief updating process (i.e., questioning existing beliefs, integrating new evidence). Future work should directly compare the role of metacognition in different types and strengths of beliefs.

Another significant finding showed that those participants who received critiquing counterevidence reported greater counterevidence-consistent belief overall. This is surprising because we adjusted participants’ pre-test belief ratings to reflect the strength of belief regardless of direction (i.e., those who initially strongly disagreed with the belief statement received the same score as those who strongly agreed). This was conducted to show the change in counterevidence-consistent belief change, given that these two groups received different counterevidence passages. However, those who initially agreed with the statement at pre-test agreed more strongly than those who disagreed. One potential concern is that these higher pre-test ratings may have minimized the amount of belief change at post-test. However, post-test belief ratings were far from ceiling, and the amount of belief change was not significantly related to the strength of belief at pre-test; thus, we are not concerned about this difference affecting our major questions of interest. Nonetheless, future studies that use a similar design may want to gather belief ratings in a pilot study to ensure that initial ratings for the two groups are equivalent.

We also assessed whether individual differences in belief updating were related to individual differences in metacognitive ability as measured by the MAI, finding no such relation. This null finding held true for participants in both the metacognition and control conditions. It is possible that the observed lack of association was due to differences in what is being manipulated and measured in the current study. The MAI is a self-report measure that asks individuals to report their perceived metacognitive ability as it relates to an educational context. In contrast, metacognitive reflection encourages the *application* of metacognitive knowledge. Put another way, the MAI may measure individuals’ trait metacognition (relatively stable individual differences in metacognitive awareness), while we were interested in manipulating participants’ state metacognition (their ability to apply metacognitive knowledge in the moment in a given situation; O’Neil and Abedi 1996). Indeed, previous work has made this distinction and found that measures of state metacognition better predicted academic achievement than measures of trait metacognition (Kosmicki 1994). Perhaps this distinction is why we failed to observe a significant relation between MAI scores and belief updating.

In addition, we found no significant relation between participants’ belief updating and their political affiliation nor the strength of their political beliefs (i.e., how extreme their political beliefs were, independent of political affiliation). Whereas previous work has found associations between political beliefs, metacognitive sensitivity, and belief updating (Rollwage et al. 2018), the topic of our study (human gene editing) involved a largely non-partisan issue without current political controversy (Gabel and Moreno 2019). In addition, some of these studies have measured radical political beliefs, like dogmatic beliefs and right-wing authoritarianism, whereas we measured political affiliation using a standard Likert scale ranging from very liberal to very conservative. Perhaps more targeted measurement of political extremism would reveal a stronger relation between political beliefs, metacognitive ability, and belief updating.

Understanding the relation between metacognitive reflection and belief updating has clear implications for educational settings (i.e., strategies for engaging with new information in a way that encourages learning). In addition, there has been a recent increase in interest in the cognitive processes behind metacognition and belief updating, likely due to the spread of misinformation about the COVID-19 pandemic and its implications for public and personal health. Indeed, a recent study showed that individuals with greater metacognitive sensitivity were more likely to change their behavior to match recommended public health measures (Fischer et al. 2021). In another recent article entitled “Research priorities for the COVID-19 pandemic and beyond: A call to action for psychological science”, one of the major calls to action involved understanding the theories and tools to promote sustained behavioral change (O’Connor et al. 2020). Other recent work has shown that brain areas involved in metacognition play a role in change-of-mind behavior (e.g., decision changes in a dot motion task or Sudoku game; Stone et al. 2022). Finally, another study showed that individuals with higher levels of metacognition were more likely to update their beliefs about climate change in the face of counterevidence (Beukelaer et al. 2023). Clearly, understanding the relation between metacognition and belief updating has implications for educating the public about factors relevant to public health and safety. This topic deserves greater attention moving forward.

### Limitations and Future Directions

The current findings provide encouraging evidence that reflecting on counterevidence can lead individuals to update their beliefs. However, these findings raise additional questions about metacognition and belief updating that cannot be adequately answered here. For example, to evaluate the effect of reflection per se, it is necessary to include a control condition in which counterevidence is presented in the absence of reflective prompts. Future work should also investigate the impact of individual differences in belief change using a measure of metacognition that measures state metacognition or the ability to apply metacognitive knowledge and skills in the moment. In particular, a behavioral measure of metacognition (e.g., confidence judgments, judgments of learning, etc.) may better capture relevant individual differences. There is also a need for more work on the relation between radical political beliefs, metacognition, and belief updating. Another limitation of the current study is that the post-test measure was administered immediately following exposure to counterevidence. Though we observed changes in participants’ beliefs across conditions, it is unclear whether these shifts in belief will persist. Future work should include follow-up belief assessments days or weeks following the intervention.

One of the main contributions of the current study is the use of a research design that allowed us to adaptively present participants with evidence counter to their existing beliefs. We chose a non-partisan topic, developed a belief statement, created supportive and critiquing counterevidence passages, and developed reflective prompts for the control and metacognition conditions. As we explained above, the political relevance of a topic may influence the interaction between metacognition and belief updating, and we believe this should be further explored in future work. In addition, we recognize that the belief statement participants read (“Scientists should be able to modify human DNA to control the expression of physical traits and risk of disease”) contained a double-barreled question. The topics of both physical traits and risk of disease were included to represent the arguments contained in both the supportive (highlighting the benefits of gene editing for preventing disease) and critiquing (highlighting the risks of modifying physical traits) counterevidence. However, future work should break this into two separate statements to more precisely measure participants’ beliefs about human gene editing. There is also great potential for exploring the role of different reflective prompts on belief updating. Perhaps some forms of metacognitive reflection (e.g., critically evaluating one’s beliefs, comparing and contrasting ideas, reflecting on one’s confidence in one’s beliefs) may have a larger impact on belief updating. We believe our experimental design can be used to systematically investigate many questions regarding metacognition and belief updating.

Finally, future work should address the fact that, while research on this topic is widespread, researchers use many different terms to refer to similar constructs. To describe metacognition, terms like metacognitive reflection, metacognitive awareness, elaborative interrogation, and others are used. In addition, belief updating (and resistance to it) is similarly referred to as belief change, conceptual change, belief perseverance, belief protection, and belief scarring, among other things. Finally, to describe counterevidence, terms like factual corrections, disconfirming evidence, counterpersuasion, persuasive refutation, and debunking are used. Given the importance of this topic, it is crucial that we consolidate relevant work and create a common language to sustain progress toward understanding the dynamics of metacognition and belief updating.

## 5. Conclusions

We demonstrated that individuals updated their belief to be more in line with presented counterevidence when asked to reflect on the counterevidence. This held true for both shallow (reflecting on the passage’s composition) and deeper reflection (reflecting on the similarities and differences between the passage and currently held beliefs). Though the interaction between time point and condition did not reach significance, there was a trend suggesting that deeper metacognitive reflection may be more likely to encourage belief change than shallower, surface level reflection. We did not find a relation between belief updating and individual differences in metacognitive ability (as measured by the MAI), political orientation, or the strength of individuals’ political beliefs. Overall, these findings show that asking individuals to deeply reflect on and provide explanations for a counter viewpoint may lead them to update their beliefs. In addition, the current study raises important questions about the relation between metacognition and belief updating and provides an experimental design to assess this relation. Understanding the relation between metacognition and belief updating has important implications for understanding how to change beliefs that may influence public health and safety.

## Figures and Tables

**Table 1:** Reflective prompts used in the control and metacognition conditions.

Control Prompts	Metacognitive Prompts
How well do you believe a non-native English speaker would understand this passage?	How consistent is this passage with your current beliefs?
Briefly write a sentence or two describing whether this passage is grammatically correct or not.	In what ways (if any) is the viewpoint in this passage similar to your own?
Please briefly describe what elements of the grammar in the passage would make it understandable to English learners.	In what ways (if any) are the viewpoints in this passage different from your own?
How well-written is this passage?	How well do you think the author of this passage argued their position?
Please briefly describe what element(s) of this passage would be hardest for English-learning individuals to comprehend.	Did this passage contain any viewpoint that was new to you?
How could the grammar in this passage be modified for English learners to make it more understandable?	What parts of the passage were compelling/convincing to you and why?

## Data Availability

The data presented in this study are openly available and can be found here: https://osf.io/cnemz/?view_only=b48ac020722741d3a627ed4a173c6142 (accessed on 30 May 2023).
